# Supplementary material for: Dysregulation of Wnt/β-catenin signaling contributes to intestinal inflammation through regulation of group 3 innate lymphoid cells
Source: Nat Commun. 2024 Apr 1;15:2820. doi: 10.1038/s41467-024-45616-1 (PMC10985070; doi:10.1038/s41467-024-45616-1)
Supplement: Supplementary file 1 — Supplementary Information [file 41467_2024_45616_MOESM1_ESM.pdf]

Supplementary Materials for

Dysregulation of Wnt/β-catenin Signaling Contributes to Intestinal Inflammation through Regulation of Group 3 Innate Lymphoid Cells

Jiacheng Hao[1,2,3,4,5], Chang Liu[2], Zhijie Gu[1,2,3,4,5], Xuanming Yang[6,7,8], Xun Lan[2,5] & Xiaohuan Guo[1,2,4]*

[1] Institute for Immunology, Tsinghua University, Beijing 100084, China.

[2] Department of Basic Medical Sciences, School of Medicine, Tsinghua University, Beijing 100084, China.

[3] School of Life Sciences, Tsinghua University, Beijing 100084, China.

[4] Beijing Key Lab for Immunological Research on Chronic Diseases, Tsinghua University, Beijing 100084, China.

[5] Tsinghua-Peking Center for Life Sciences, Tsinghua University, Beijing, China.

[6] Sheng Yushou Center of Cell Biology and Immunology, School of Life Sciences and Biotechnology, Shanghai Jiao Tong University, Shanghai 200240, China.

[7] Joint International Research Laboratory of Metabolic and Developmental Sciences, Shanghai Jiao Tong University, Shanghai 200240, China.

[8] Key Laboratory of Systems Biomedicine (Ministry of Education), Shanghai Center for Systems Biomedicine, Shanghai Jiao Tong University, Shanghai 200240, China.

* Correspondence: guoxiaohuan@tsinghua.edu.cn

This PDF file includes:

Supplementary Figures. 1-7
Supplementary Table. 1

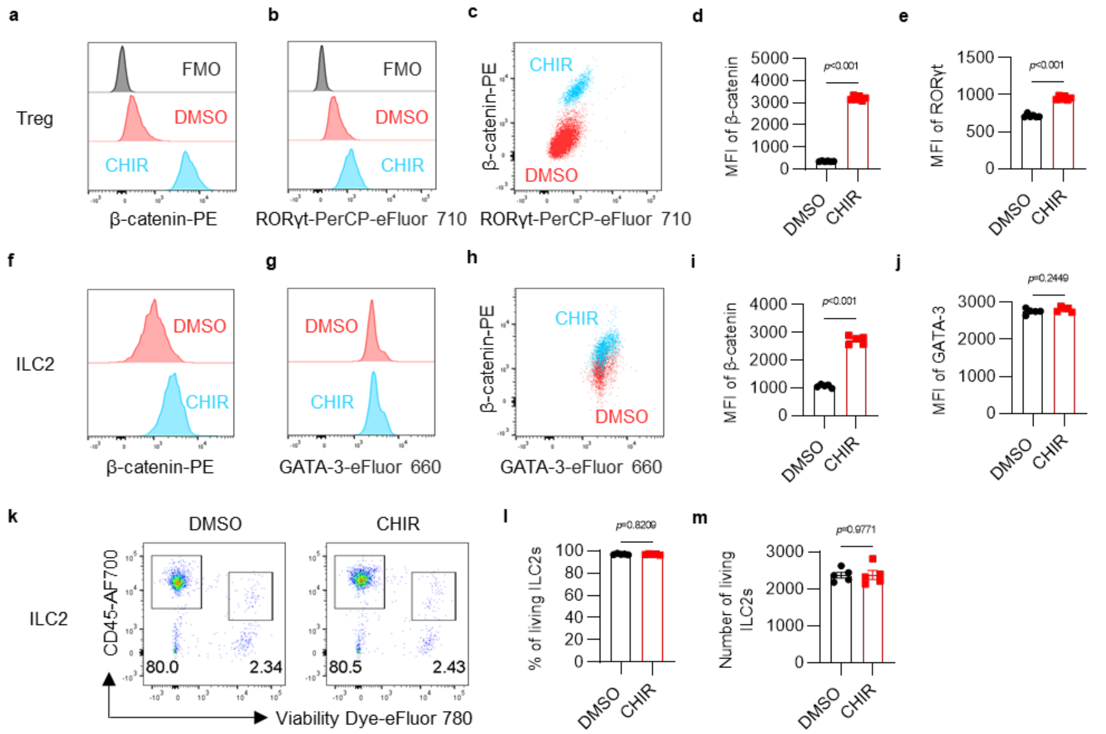

Supplementary Fig. 1. Activation of Wnt/β-catenin signaling differentially affects various immune cells *in vitro*.

(**a-e**) Naïve T cells (gated in $7AAD^{-}CD4^{+}CD25^{-}CD62L^{high}CD44^{low}$) from wild type mice were sorted and Treg cells were induced *in vitro*. Induced Treg cells were treated with DMSO and CHIR-99021 (CHIR) for 96 hours. Flow cytometry analysis of β-catenin (**a**) and RORγt (**b**) are shown (**c**). FMO represents fluorescence minus one control. Mean fluorescence intensity (MFI) of β-catenin (**d**) and RORγt (**e**) are shown. (**f-m**) ILC2s (gated in $7AAD^{-}lineage^{-}CD127^{+}KLRG1^{+}$) from *Rag1*$^{-/-}$ mice were treated with DMSO and CHIR-99021 (CHIR) for 24 hours *in vitro*. Flow cytometry analysis of β-catenin (**f**) and RORγt (**g**) are shown (**h**). Mean fluorescence intensity (MFI) of β-catenin (**i**) and RORγt (**j**) are shown. Flow cytometry analysis of cell viability (**k**), the percentage and number of living ILC2s (**l**, **m**) are shown.

Each dot represents one individual replicate (n = 6 per group in **d**, **e**, n = 5 per group in **i**, **j**, **l**, **m**). Error bars represent the SEM. Statistical significance was tested by unpaired two-sided Student's *t*-test. Data are representative of three independent experiments.

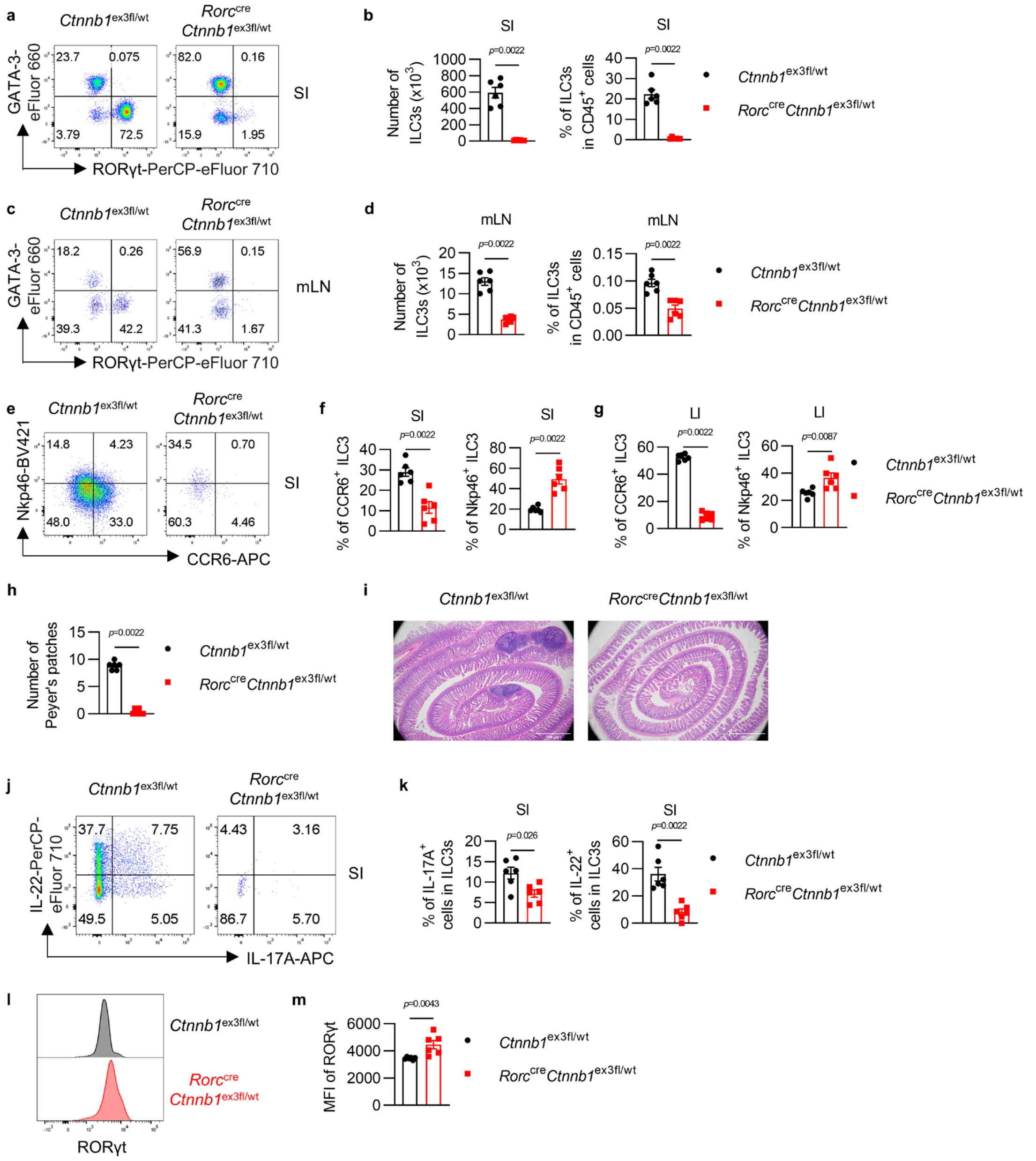

Supplementary Fig. 2. Activation of β-catenin differentially affects ILC3s and thymocytes *in vivo*.

The naive *Ctnnb1*$^{ex3fl/wt}$ and *Rorc*^{cre}*Ctnnb1*$^{ex3fl/wt}$ mice were euthanized and analyzed at 8-week-old. (**a**) Flow cytometry analysis of ILCs (gated in lineage^{-}CD127^{+}) in small intestine (SI). (**b**) The number and percentage of ILC3s in total CD45^{+} cells in SI. (**c**) Flow cytometry analysis of ILCs (gated in lineage^{-}CD127^{+}) in mesenteric lymph nodes (mLN). (**d**) The number and percentage of ILC3s in total CD45^{+} cells in mLN. (**e**) Flow cytometry analysis of ILC3 subsets (gated in lineage^{-}CD127^{+}RORγt^{+}) in SI. (**f**) The proportion of CCR6^{+} and Nkp46^{+} ILC3s in SI. (**g**) The proportion of CCR6^{+} and

Nkp46^{+} ILC3s in large intestine (LI). (**h**) The number of Peyer's patches in SI. (**i**) Histological analysis of representative small intestines from *Ctnnb1*$^{ex3fl/wt}$ and *Rorc*^{cre}*Ctnnb1*$^{ex3fl/wt}$ mice. Scale bars represent 500 μm. (**j**) Flow cytometry analysis of cytokine production in ILC3s (gated in lineage^{-}CD127^{+}RORγt^{+}) in SI. Cytokine production was detected under PMA/Ionomycin treatment. (**k**) The percentage of IL-17A^{+} and IL-22^{+} cells in total ILC3s in SI. (**l**) Flow cytometry analysis of RORγt expression in CD4^{+}CD8^{+} thymocytes. (**m**) Mean fluorescence intensity (MFI) of RORγt in CD4^{+}CD8^{+} thymocytes.

Each dot represents one individual mouse (n = 6 per group). Error bars represent the SEM. Statistical significance was tested by unpaired two-sided Mann-Whitney U test. Data are representative of three independent experiments.

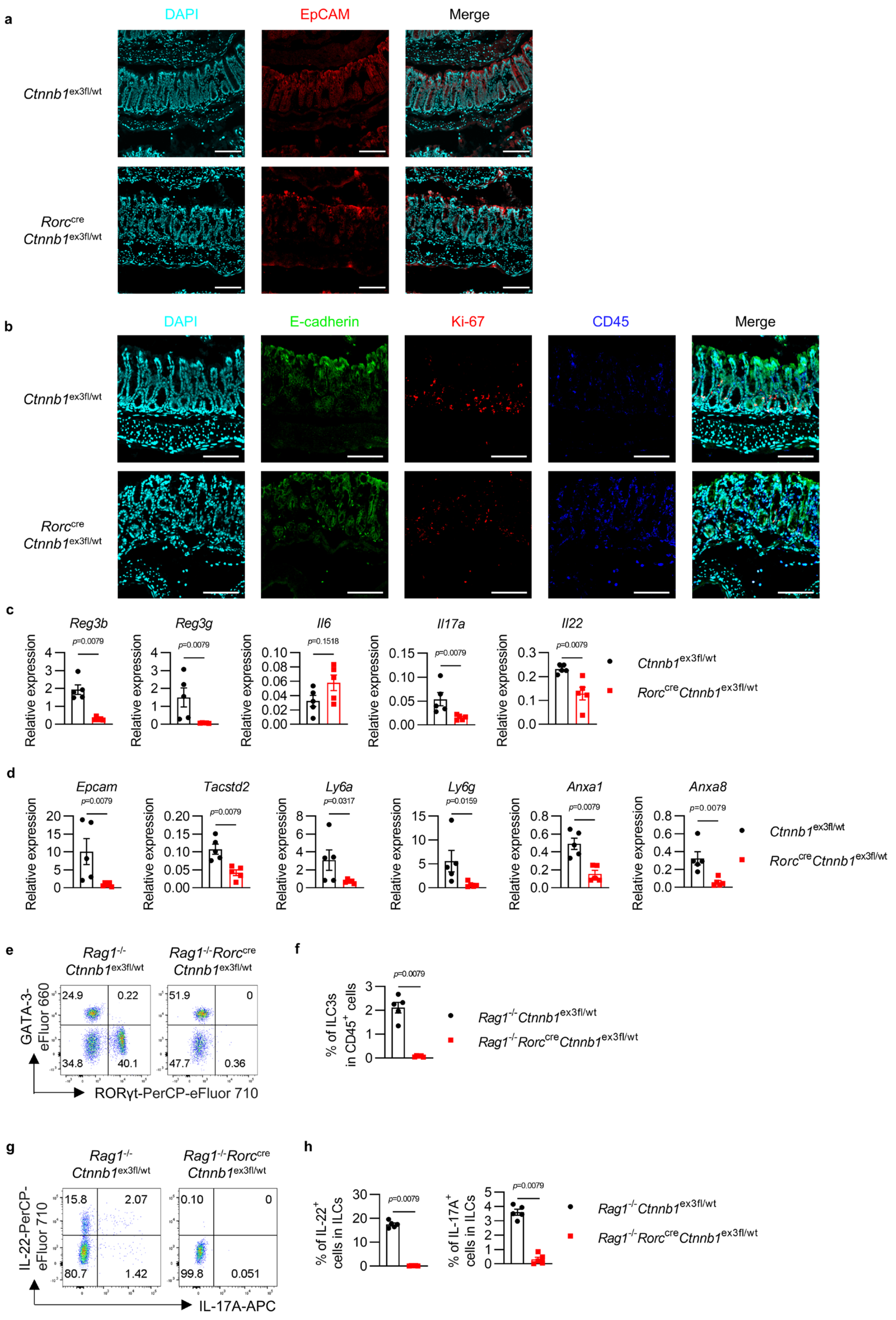

Supplementary Fig. 3. Activated Wnt/β-catenin signaling in ILC3s exacerbates DSS-induced colitis.

(**a-d**) 8-week-old *Rorc*^{cre}*Ctnnb1*$^{ex3fl/wt}$ mice (n = 5) and their littermates *Ctnnb1*$^{ex3fl/wt}$

mice (n = 5) were given 3% DSS in drinking water for 5 days, followed by normal drinking water. The epithelium integrity, proliferation of intestinal epithelial cells and immune cell infiltration were visualized by Immunofluorescence (IF) staining (**a**, **b**) Scale bars represent 100 μm. The relative expression of indicated genes in the colon were detected by qPCR, normalized to *Gapdh* (**c**, **d**).

(**e-h**) 8-week-old *Rag1*$^{-/-}$*Rorc*^{cre}*Ctnnb1*$^{ex3fl/wt}$ mice (n = 5) and their littermates *Rag1*$^{-/-}$*Ctnnb1*$^{ex3fl/wt}$ mice (n = 5) were given 3% DSS in drinking water for 5 days, followed by normal drinking water. (**e**) Flow cytometry analysis of colonic ILCs (gated in lineage^{-}CD127^{+}). (**f**) The percentage of colonic ILC3s in total CD45^{+} cells. (**g**) Flow cytometry analysis of cytokine production in colonic ILCs (gated in lineage^{-}CD127^{+}). Cytokine production was detected under PMA/Ionomycin treatment. (**h**) The percentage of IL-17A^{+} and IL-22^{+} cells in total colonic ILCs.

Each dot represents one individual mouse (n = 5). Error bars represent the SEM. Statistical significance was tested by unpaired two-sided Mann-Whitney U test. Data are representative of two (**a**, **b**, **e-h**) or three (**c**, **d**) independent experiments.

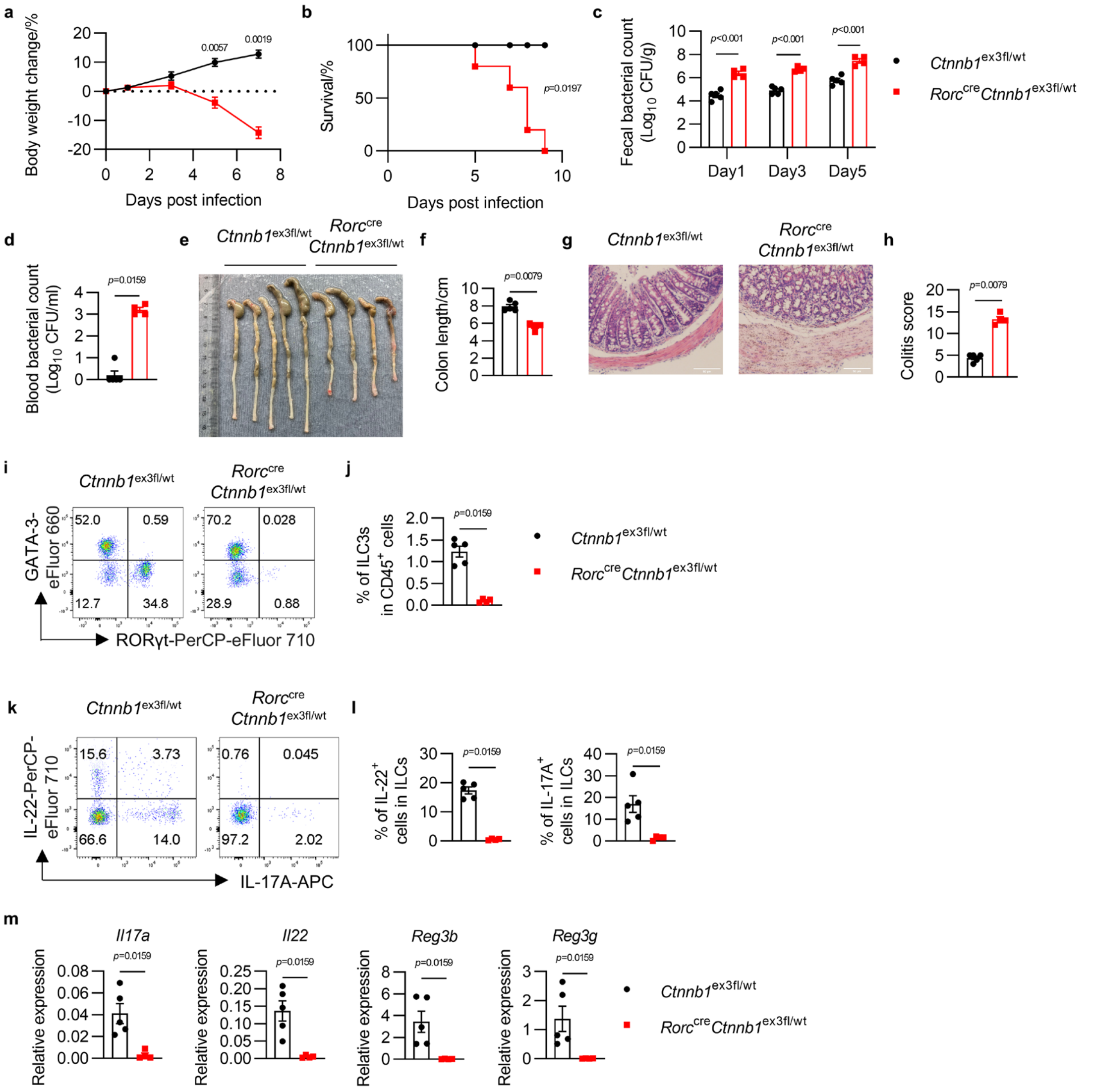

Supplementary Fig. 4. Activated Wnt/β-catenin signaling in ILC3s impairs host defense against *C. rodentium* infection.

8-week-old *Rorc*^{cre}*Ctnnb1*$^{ex3fl/wt}$ mice and their littermates *Ctnnb1*$^{ex3fl/wt}$ mice were orally inoculated with *C. rodentium* (2×10^9 CFU). (**a-b**) Body weight change (**a**) and survival rates (**b**) at different time points (n = 5 per group). (**c**) Bacterial titers from fecal homogenate at indicated days. (**d**) Bacterial titers from blood homogenate at day 5 post infection. (**e-f**) Gross morphological changes (**e**) and lengths of colons (**f**). (**g**) Histological analysis of representative colons. Scale bars represent 50 μm. (**h**) Colitis score of *Ctnnb1*$^{ex3fl/wt}$ and *Rorc*^{cre}*Ctnnb1*$^{ex3fl/wt}$ mice. (**i**) Flow cytometry analysis of colonic ILCs (gated in lineage^{-}CD127^{+}). (**j**) The percentage of colonic ILC3s in total CD45^{+} cells. (**k**) Flow cytometry analysis of cytokine production in colonic ILCs (gated

in lineage$^-$CD127$^+$). Cytokine production was detected under PMA/Ionomycin treatment. (**l**) The percentage of IL-17A$^+$ and IL-22$^+$ cells in total colonic ILCs. (**m**) The relative expression of indicated genes in the colon were detected by qPCR, normalized to *Gapdh*.

Each dot represents one individual mouse (for **a** and **b**, n = 5 per group, for **c** to **m**, *Ctnnb1*$^{ex3fl/wt}$ mice n = 5, *Rorc*^{cre}*Ctnnb1*$^{ex3fl/wt}$ mice n = 4). Error bars represent the SEM. Statistical significance was tested by two-sided two-way ANOVA with Sidak correction adjusted for multiple comparisons (**a**), two-sided log-rank Mantel-Cox test (**b**), two-sided one-way ANOVA with Tukey's test adjusted for multiple comparisons (**c**) and unpaired two-sided Mann-Whitney *U* test (**d**, **f**, **h**, **j**, **l**, **m**). Data are representative of three independent experiments.

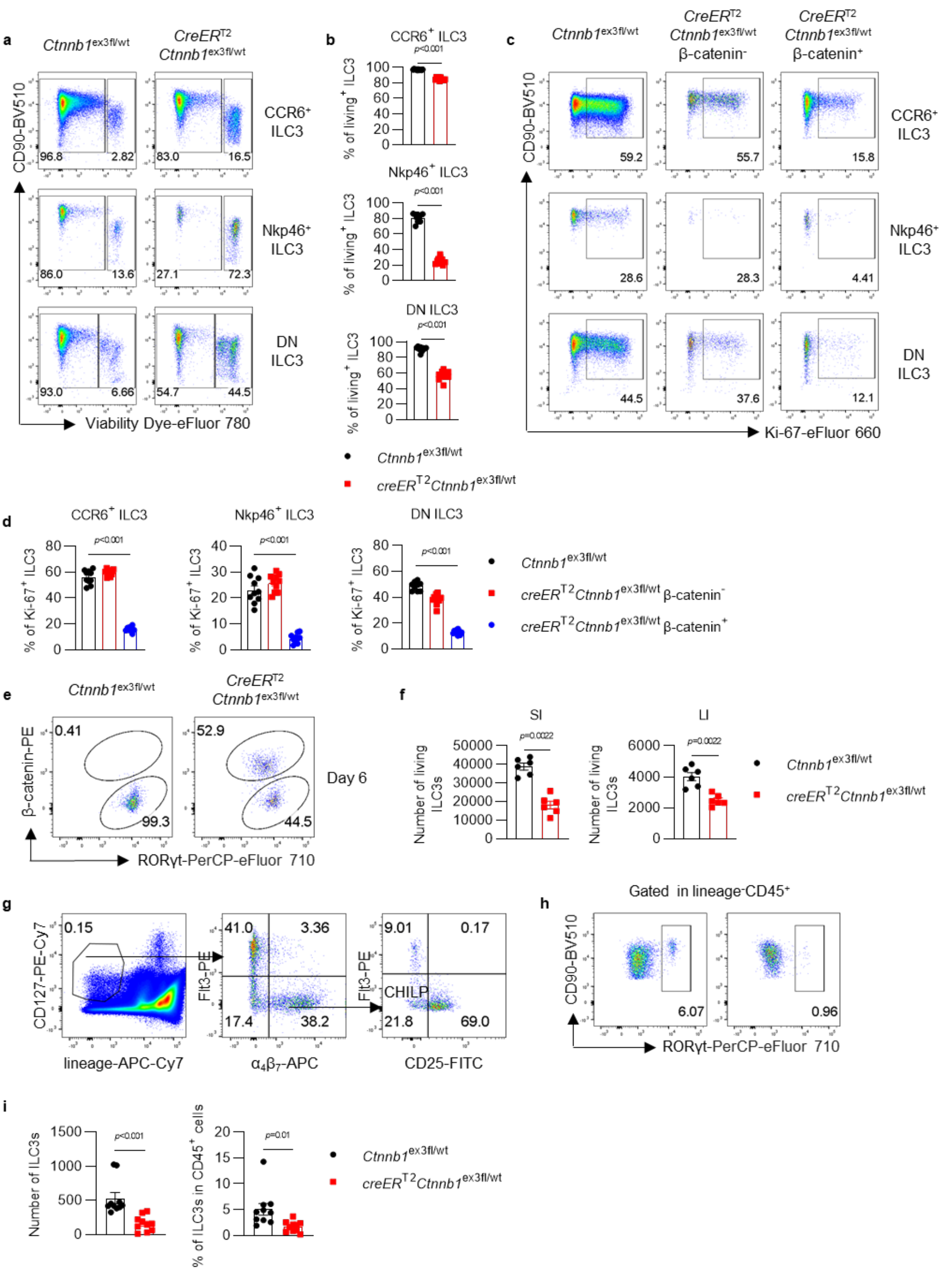

Supplementary Fig. 5: Activated β-catenin signaling inhibits ILC3 proliferation and survival.

(**a-d**) CCR6^{+}, Nkp46^{+} and CCR6/Nkp46 double negative (DN) ILC3 subsets (gated in 7AAD^{-}lineage^{-}CD90^{high}CD45^{low}) from *Ctnnb1*$^{ex3fl/wt}$ mice and *CreER*^{T2}*Ctnnb1*$^{ex3fl/wt}$ mice were sorted separately by flow cytometry, then were cultured *in vitro* with OP9 feeder cells, treated with 4-hydroxytamoxifen at day 0 and detected by flow cytometry

at day 9. Cells from *CreER*^{T2}*Ctnnb1*$^{ex3fl/wt}$ mice were further divided into two subsets: β-catenin^{-} ILC3s and β-catenin^{+} ILC3s. (**a**) Flow cytometry analysis of cell viability. (**b**) The percentage of living ILC3s. (**c**) Flow cytometry analysis of Ki-67^{+} ILC3s. (**d**) The percentage of Ki-67^{+} ILC3s in different groups at day 9.

(**e-f**) ILC3s (gated in 7AAD^{-}lineage^{-}CD90^{high}CD45^{low}) from *Ctnnb1*$^{ex3fl/wt}$ mice and *CreER*^{T2}*Ctnnb1*$^{ex3fl/wt}$ mice were isolated and transferred into *Rag2*$^{-/-}$*Il2rg*$^{-/-}$ recipients (*n* = 6 per group) respectively, and then treated with tamoxifen for 5 days to activate Wnt pathway in ILC3 *in vivo*. (**e**) Flow cytometry analysis of RORγt and β-catenin expression in ILC3 from gut at day 6. (**f**) The number of total ILC3s in small intestine (SI) and large intestine (LI) at day 9.

(**g-i**) Common helper ILC progenitors (CHILPs) from *Ctnnb1*$^{ex3fl/wt}$ mice and *CreER*^{T2}*Ctnnb1*$^{ex3fl/wt}$ mice were isolated and cultured *in vitro* with OP9 feeder cells. Cells were treated with 4-hydroxytamoxifen at day 0 and the cells were analyzed at day 9. (**g**) Gating strategy of CHILPs (7AAD^{-}lineage^{-}CD127^{+}$\alpha_4\beta_7^{+}$Flt3^{-}CD25^{-}). (**h**) Flow cytometry analysis of RORγt^{+} ILC3s (gated in lineage^{-}CD45^{+}). (**i**) The number and percentage of RORγt^{+} ILC3s in total CD45^{+} cells.

Each dot represents one individual replicate (**b**, **d**, **i**, *n* = 10) or mouse (**f**, *n* = 6). Error bars represent the SEM. Statistical significance was tested by unpaired two-sided Student's *t*-test (**b**, **i**), two-sided one-way ANOVA with Tukey's test adjusted for multiple comparisons (**d**) and unpaired two-sided Mann–Whitney *U* test (**f**). Data are representative of two (**e**, **f**) or three (**a-d**, **g-i**) independent experiments.

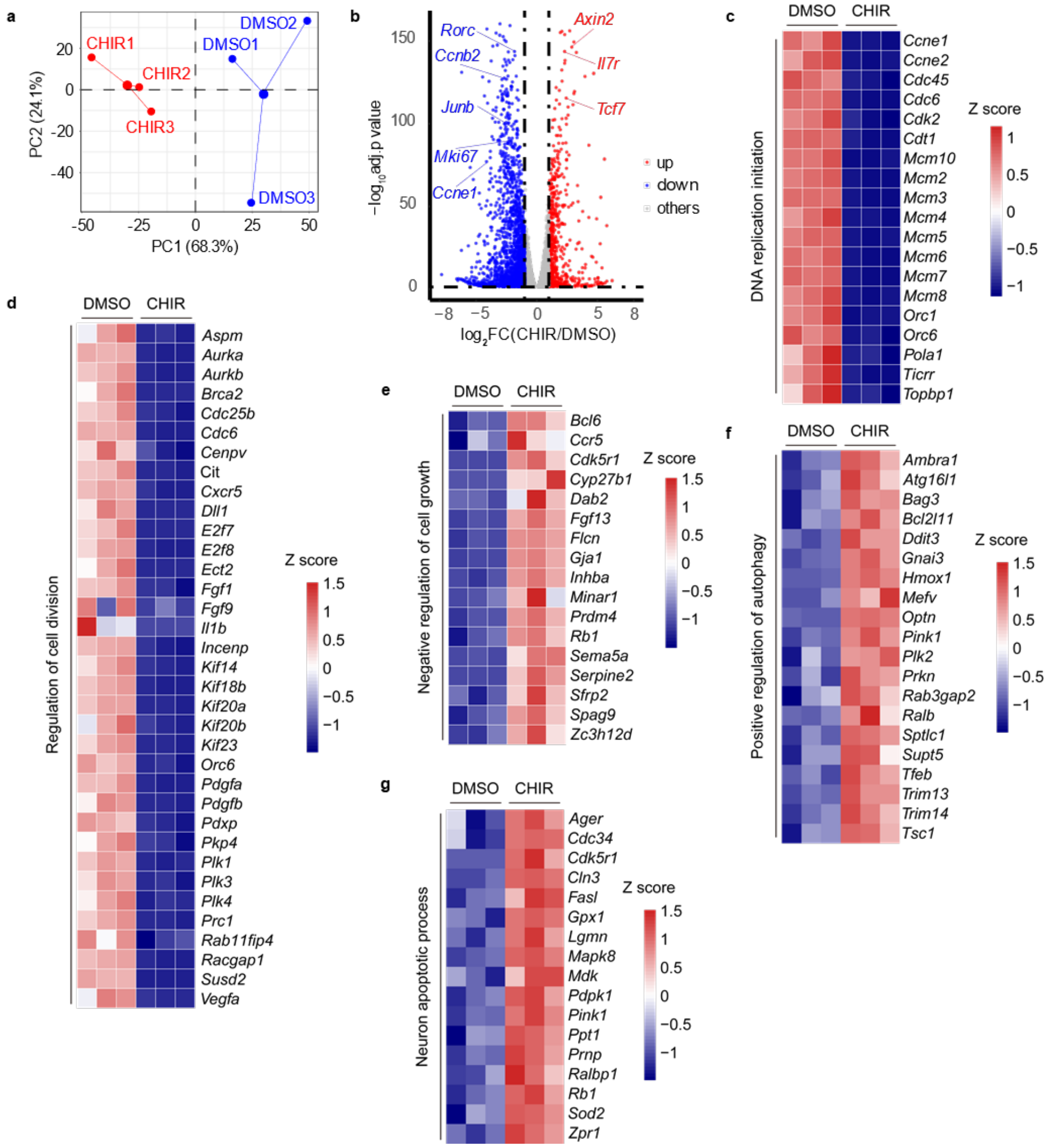

Supplementary Fig. 6. The activation of Wnt/β-catenin signaling influences the cell proliferation of ILC3s.

ILC3s from *Rag1*$^{-/-}$ mice were treated with DMSO and CHIR-99021 (CHIR) for 24 hours *in vitro*, and performed with RNA-seq. (**a**) PCA of RNA-seq performed on DMSO-treated and CHIR-treated ILC3s. (**b**) Volcano plot of differentially expressed genes in CHIR-treated ILC3s. Red represents upregulating genes in CHIR-treated ILC3s, blue represents downregulating genes in CHIR-treated ILC3s. (**c-g**) Heatmap of normalized counts comparing gene expression for transcripts related to DNA replication initiation (**c**), regulation of cell division (**d**), negative regulation of cell growth (**e**), positive regulation of autophagy (**f**) and neuron apoptotic process (**g**).

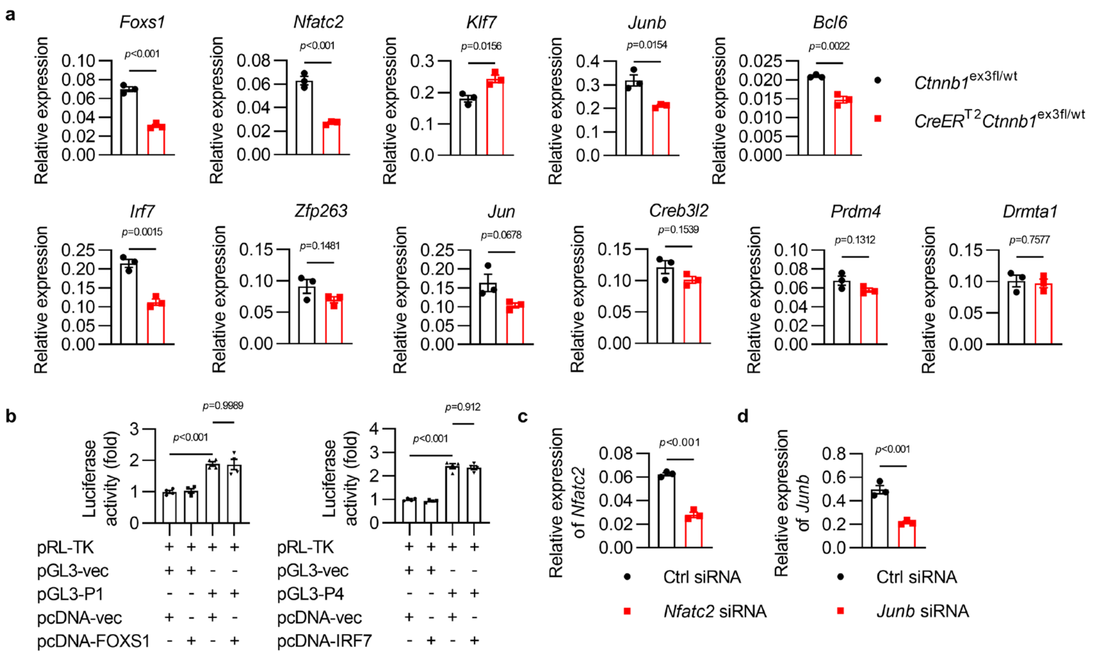

Supplementary Fig. 7. Dysregulation of Wnt/β-catenin signaling in ILC3 inhibits NFATc2 and JunB expression.

(**a**) ILC3s from *Ctnnb1*$^{\text{ex3fl/wt}}$ mice and *CreER*$^{\text{T2}}$*Ctnnb1*$^{\text{ex3fl/wt}}$ mice were treated with 4-hydroxytamoxifen. Relative mRNA expression of indicated genes are shown.

(**b**) Luciferase activity in 293T cells transfected with vector alone (pGL3-vec) or vector containing *Rorc* locus P1 or P4 (pGL3-P1 or pGL3-P4), together with empty vector (pcDNA-vec) or vector expressing FOXS1 (pcDNA-FOXS1) and IRF7 (pcDNA-IRF7). The results represent the relative firefly luciferase activities normalized to the corresponding Renilla luciferase activities.

(**c-d**) Relative mRNA expression of *Nfatc2* (**c**) or *Junb* (**d**) in ILC3s transfected with Ctrl siRNA, *Nfatc2* siRNA or *Junb* siRNA.

Each dot represents one individual replicate (n = 3 in **a, c, d,** n = 4 in **b**). Error bars represent the SEM. Statistical significance was tested by unpaired two-sided Student's *t*-test (**a**, **c**, **d**) and two-sided one-way ANOVA with Tukey's adjusted test for multiple comparisons (**b**). Data are representative of three independent experiments.

Supplementary Table 1. Primer list

Gene symbol	Forward primer sequence	Reverse primer sequence
Gata3	CTCGGCCATTCGTACATGGAA	GGATACCTCTGCACCGTAGC
Id2	CGACTGCTACTCCAAGCTCAA	CCACAGAGTACTTTGCTATCAT
Maf	GGAGACCGACCGCATCATC	TCATCCAGTAGTAGTCTTCCAGG
Maff	ACCTGTCGGATGAAGCGCTGAT	TAGCCGCGGTTCTTGAGTGTGC
Nr4a1	TTGAGTTCGGCAAGCCTACC	GTGTACCCGTCCATGAAGGTG
Rara	TTCTTTCCCCCTATGCTGGGT	GGGAGGGCTGGGTACTATCTC
Rorc	TACCTTGGCCAAAACAGAGG	ATGCCTGGTTTCCTCAAAA
Stat3	CAATACCATTGACCTGCCGAT	GAGCGACTCAAACTGCCCT
Tbx21	GTGAAGGACAGGAATGGGAA	GGTGTCTGGGAAGCTGAGAG
Tox2	CTGGGCTTCCGTGGAATGAG	CACGTAGGCACTGTCACCATC
Nfil3	ACCTAGCCTCAGGTGAAGATT	AGGGAGAGCAGCTCAGCTTT
Rora	GTGGAGACAAATCGTCAGGAAT	TGGTCCGATCAATCAAACAGTTC
Tcf7	GAGCCTGCAGGCCTTCAAG	GATGCAAGTTGCCAGGCTGG
Cxcr5	ATGAACTACCCACTAACCCTGG	TGTAGGGGAATCTCCGTGCT
H2-Ab1	AGCCCCATCACTGTGGAGT	GATGCCGCTCAACATCTTGC
H2-K1	CGTTCCAGGGGATGTACGG	GCTCCCACTTGTGTTTGGTGA
Il2rb	TGGAGCCTGTCCCTCTACG	TCCACATGCAAGAGACATTGG
Il2rg	CTCAGGCAACCAACCTCAC	GCTGGACAACAAATGTCTGGTAG
Ltb	TGGCAGGAGCTACTTCCCT	TCCAGTCTTTTCTGAGCCTGT
Ncr1	ATGCTGCCAACACTCACTG	GATGTTCACCGAGTTTCCATTTG
Cd4	AGGTGATGGGACCTACCTCTC	GGGGCCACCACTTGAACTAC
Il1r1	GGGAAGCAATATCCGGTCACA	TGACGTTGCAGATCAGTTGTATC
Il23r	TTCAGATGGGCATGAATGTTTCT	CCAAATCCGAGCTGTTGTTCTAT
Il7r	GCGGACGATCACTCCTTCTG	AGCCCCACATATTTGAAATTCCA
Csf2	GGCCTTGGAAGCATGTAGAGG	GGAGAACTCGTTAGAGACGACTT
Il2	CGCAGAGGTCCAAGTTCATC	AACTCCCCAGGATGCTCAC
Vegfa	CTGCCGTCCGATTGAGACC	CCCCTCCTTGTACCACTGTC
Blk	GAGGCAGGTCAGTGAGAAGG	GTCCTGGTTAGGAGATGGTGG
Ccl17	TACCATGAGGTCACTTCAGATGC	GCACTCTCGGCCTACATTGG
Ccne1	GTGGCTCCGACCTTTCAGTC	CACAGTCTTGTCAATCTTGGCA
Cdk1	AGAAGGTACTTACGGTGTGGT	GAGAGATTTCCCGAATTGCAGT
Cdk19	GGTCAAGCCTGACAGCAAAGT	TTCCTGGAAGTAAGGGTCCTG
Cx3cl1	ACGAAATGCGAAATCATGTGC	CTGTGTCGTCTCCAGGACAA
Cxcr4	GAAGTGGGGTCTGGAGACTAT	TTGCCGACTATGCCAGTCAAG
Ffar2	CTTGATCCTCACGGCCTACAT	CCAGGGTCAGATTAAGCAGGAG
Ikzf3	CTGAATGACTACAGCTTGCCC	GCTCCGGCTTCATAATGTTCT
Il1r2	GTTTCTGCTTTCACCACTCCA	GAGTCCAATTTACTCCAGGTCAG
Irf4	TCCGACAGTGGTTGATCGAC	CCTCACGATTGTAGTCCTGCTT
Lef1	TGTTTATCCCATCACGGGTGG	CATGGAAGTGTCGCCTGACAG
Slc6a7	ACCTGGATGTAGACTTCGCAG	CGCCAGACATTTCCCAAGC

Cd69	CCCTTGGGCTGTGTTAATAGTG	AACTTCTCGTACAAGCCTGGG
Fos	CGGGTTTCAACGCCGACTA	TTGGCACTAGAGACGGACAGA
Fosb	TTTTCCCGGAGACTACGACTC	GTGATTGCGGTGACCGTTG
Il15ra	CGTGTCCACCTCCCGTATCTA	AGACATACCTCTCCCTGGAGT
Il7	TTCCTCCACTGATCCTTGTTCT	AGCAGCTTCCTTTGTATCATCAC
Mki67	ATCATTGACCGCTCCTTTAGGT	GCTCGCCTTGATGGTTCCT
Nfatc2	CTCGGCCTTTGCCCATCTC	AGGAGCACGGAGCATCTGA
Junb	TCACGACGACTCTTACGCAG	CCTTGAGACCCCGATAGGGA
Reg3b	ATGGCTCCTACTGCTATGCC	GTGTCCTCCAGGCCTCTT
Reg3g	CAAGGTGAAGTTGCCAAGAA	CCTCTGTTGGGTTCATAGCC
Il6	ACCAGAGGAAATTTTCAATAGGC	TGATGCACTTGCAGAAAACA
Il17a	TTTAACTCCCTTGGCGCAAAA	CTTTCCCTCCGCATTGACAC
Il22	CATGCAGGAGGTGGTACCTT	CAGACGCAAGCATTTCTCAG
Epcam	GCGGCTCAGAGAGACTGTG	CCAAGCATTTAGACGCCAGTTT
Tacstd2	AGGGGCTTGGATCTAGCAC	TTGCCCGACATTGGCAGAC
Ly6a	AGGAGGCAGCAGTTATTGTGG	CGTTGACCTTAGTACCCAGGA
Ly6g	GACTTCCTGCAACACAACTACC	ACAGCATTACCAGTGATCTCAGT
Anxa1	ATGTATCCTCGGATGTTGCTGC	TGAGCATTGGTCCTCTTGGTA
Anxa8	GAGCTGCACGATGCCATGA	CGGTTCCCATAATCTTCTCACC
Foxs1	CTATCCAGAGTTCACCGGGTC	GTTATGGCGGATGCTGTTTTG
Klf7	TCCACGACACCGGCTACTT	GGGAGCAGCAAGGGGTCTA
Bcl6	GATACAGCTGTCAGCCGGG	AGTTTCTAGGAAAGGCCGGA
Irf7	GAGACTGGCTATTGGGGGAG	GACCGAAATGCTTCCAGGG
Zfp263	CGTATGCAGAAAGAACTTGGGA	CAGGGCTTCGCTCAGTCTC
Jun	CCTTCTACGACGATGCCCTC	GGTTCAAGGTCATGCTCTGTTT
Creb3l2	CATGTACCACACGCACTTCTC	CCACCTCCATTGACTCGCTC
Prdm4	TTCAGAACCATTCTACCTGGCA	ATACTGAGGAGGTGGTCTGTC
Drmta1	CCCAACTTTCGAGGTTTTCCA	CCCAGAGAATGGTGATGAGTGTT
